# Down-regulated miR-448 relieves spinal cord ischemia/reperfusion injury by up-regulating SIRT1

**DOI:** 10.1590/1414-431X20177319

**Published:** 2018-03-15

**Authors:** Yun Wang, Qing-Jiang Pang, Jiang-Tao Liu, Hai-Hao Wu, Dong-Ying Tao

**Affiliations:** 1 Department of Orthopedics, Ningbo No. 2 Hospital, Ningbo, Zhejiang, China; 2 Department of Human Morphology, Ningbo College of Health Sciences, Ningbo, Zhejiang, China

**Keywords:** SCII, miR-448, SIRT1, Nerve cells

## Abstract

MicroRNAs play a crucial role in the progression of spinal cord ischemia/reperfusion injury (SCII). The role of miR-448 and SIRT1 in SCII was investigated in this study, to provide further insights into prevention and improvement of this disorder. In this study, expressions of miR-448 and SIRT1 protein were determined by qRT-PCR and western blot, respectively. Flow cytometry was used to analyze cell apoptosis. The endogenous expression of genes was modulated by recombinant plasmids and cell transfection. Dual-luciferase reporter assay was performed to determine the interaction between miR-448 and SIRT1. The Basso, Beattie, and Bresnahan score was used to measure the hind-limb function of rat. The spinal cord ischemia reperfusion injury model of adult rats was developed by abdominal aorta clamping, and the nerve function evaluation was completed by motor deficit index score. In SCII tissues and cells treated with hypoxia, miR-448 was up-regulated while SIRT1 was down-regulated. Hypoxia treatment reduced the expression of SIRT1 through up-regulating miR-448 in nerve cells. Up-regulation of miR-448 induced by hypoxia promoted apoptosis of nerve cells through down-regulating SIRT1. Down-regulated miR-448 improved neurological function and hind-limb motor function of rats with SCII by up-regulating SIRT1. Down-regulated miR-448 inhibited apoptosis of nerve cells and improved neurological function by up-regulating SIRT1, which contributes to relieving SCII.

## Introduction

Spinal cord injury (SCI) begins with neurological damage in the spinal cord and results in devastating deficits in sensorimotor functions and other complications (1). Paraplegia and tetraplegia are the most common and serious disabilities caused by SCI, causing grave impairment in patients (2,3). Spinal cord ischemia/reperfusion injury (SCII) is a serious complication of thoracoabdominal aortic surgery, such as abdominal aortic aneurism surgery resection (4). SCII also leads to many complications, including paraplegia, and its prevention, treatment, and rehabilitation have attracted increasing attention.

Sirtuin refers to a protein family that is homologous with the silent information regulation 2 (Sir2), and Sirtuin 1 (SIRT1) is the closest mammalian homologue of the yeast Sir2 that linked to neurodegenerative diseases (5). A previous study has indicated that overexpression of SIRT1 protein in neurons protected against experimental autoimmune encephalomyelitis (EAE) by activating multiple SIRT1 targets (6). In addition, activation of SIRT1 by resveratrol contributed to alleviating neuropathic pain in rat model and played a key role in protecting spinal astrocytes (7,8). All the above findings demonstrated the crucial neuro-protective effect of SIRT1 in neurological diseases.

MicroRNAs (miRNAs) are a class of noncoding RNAs that negatively regulate gene expression at the post-transcriptional level (9). Altered expression of various miRNAs following a traumatic SCI has been identified in adult rats (10). Bioinformatics analysis showed the complementary base pairs between SIRT1 and miR-448, suggesting the potential binding sites between them. It has been reported that miR-448 was greatly up-regulated in rat hippocampus following chronic lead exposure, which may be associated with neurophysiological pathways and neurodegenerative diseases (11). However, so far, the influence of miR-448 on SCII has not been reported.

In consideration of the potential binding sites between SIRT1 and miR-448, and their roles in neurological disorders, we hypothesized that they might interact with each other and have an impact on SCII progression. Therefore, the expression levels and interplay of miR-448 and SIRT1 in SCII were evaluated in this study, to explore their role in SCII pathogenesis and treatment.

## Material and Methods

### Animal preparation

The animal study was approved by the Animal Care Committee of the Ningbo No. 2 Hospital. All protocols of animal experiments were performed according to the Guide for the Care and Use of Laboratory Animals by the National Institutes of Health.

Adult male Sprague-Dawley rats weighing 250–320 g were used in this study and were randomly divided into sham-operation control group (n=12) and SCII experimental group (SCII, n=12). All rats were kept in standard conditions and given access to food and water *ad libitum*, and none of them had any neurological disorder before operation.

### Establishment of SCII model

Rats of the SCII experimental group (SCII, n=12) were anesthetized with 10% chloral hydrate (0.35 mL/100 g) through intraperitoneal injection. The maintenance of anesthesia was achieved by injection with half of the quantity (0.17 mL/100 g) each hour throughout the experiment. Rats were shaved and aseptically treated to construct the SCII model as previous description (12). In supine position on the operation plate and with the surgical area cleaned, a standard midline laparotomy was performed and the abdominal aorta was exposed. After identification of the bilateral renal artery, the spinal cord ischemia was done by clamping the abdominal aorta with a bulldog clamp. After occlusion, abdominal artery pulsation ceased, and blood flow was obstructed for 40 min. Disappearance of pulse of the lower abdominal artery meant success of clamping. The bulldog clamp was removed, and the abdominal wall was closed. A successful rat model of spinal cord ischemia criterion is cessation of abdominal aorta pulse and double hind limb skin cyanosis. Once the arterial clamp was loosened, the pulse of the abdominal aorta recurred and the skin of both hind limbs became bright red, which meant recanalization of blood flow. For the control group (n=12), laparotomy was performed in the same way but without abdominal aorta clamping. The anterior abdominal wall was sutured with polypropylene suture in all rats at the end of surgery.

### BBB scoring

Hind limb motor function assessment was performed at 7 days post-surgery by using the Basso, Beattie, and Bresnahan (BBB) motor rating scale (13). The BBB scale was based on motor ability following SCII in a rat model. BBB scores reflect a 21-point open field locomotor scale, where 0 indicates no locomotion and 21 normal motor functions. Rats' hind limb movements, trunk position, stability, stepping, coordination, paw placement, toe clearance, and tail position were analyzed during the evaluation period. Two blind observers evaluated the scores individually, and the mean value of the two observers' scores was used.

### Cell culture and hypoxia treatment

Nerve cell lines including AGE1.HN and PC12 cells were cultured in DMEM medium (Gibco, USA) supplemented with 10% fetal bovine serum (FBS, Gibco), 100 U/mL penicillin and 100 µg/mL streptomycin (Heclony, USA), and kept at 37°C in a humidified atmosphere with 5% CO_2_. The hypoxia treatment was achieved by oxygen-glucose deprivation (OGD) described in a previous study (14). If brief, the medium DMEM without glucose (Gibco) was placed in the Ruskin Bug Box Plus (Ruskinn Technology Ltd., UK) humidified airtight hypoxic chamber for 2 h to maintain an environment of 95% N_2_/5%CO_2_ at 37°C and verified with Anaerobic Indicator (Oxoid Ltd., UK). The maintenance culture medium was removed, cells washed with PBS, and experimental hypoxic medium was added to the cell culture wells. OGD was induced by placing the plates in the hypoxic chamber. With OGD completed, cells were returned to a normal incubator for reperfusion and OGD media were replaced with normal DMEM medium.

### RNA extraction and quantitative real-time PCR (qRT-PCR)

Spinal cord segments between L4 and L6 were obtained from the operated spinal cord area of the SCII rat model, and divided into three (5 mm) equal parts for detection of gene expressions (15). Trizol reagent (Invitrogen, USA) was used to extract total RNA from spinal cord tissues and nerve cells according to the instruction of the manufacturer. The cDNA Synthesis Kit (Invitrogen) was used to preform reverse transcription of total RNA to synthesize cDNA, and the qRT-PCR was performed with SYBR Green Master Mix (ABI, USA) on an ABI Prism 7000 Sequence Detection. Primers were provided by Sangon Biotech (China). The U6 and GAPDH were used as control. The 2^-ΔΔCt^ method was used to analyze the relative expression of miR-448 and SIRT1 mRNA.

### Western blot analysis

Tissues and cells were treated with lysis buffer (Beyotime Biotechnology, China), and the lysates was centrifuged to get the total protein. Protein concentration was determined with a BCA protein assay kit (Pierce, USA). Then, equal amounts of protein were separated by 10% SDS-PAGE with an electrophoresis system (Bio-Rad, USA) and transferred into the polyvinylidene difluoride (PVDF) membranes (Invitrogen). The membranes were then blocked for 1 h with 5% skimmed milk at room temperature, and then incubated overnight with primary antibodies including anti-SIRT1 antibody (1:1200, Abcam, UK) and anti-β-actin antibody (Abcam, 1:2000) at 4°C. The membrane was incubated with HRP-bounded antibodies for 1 h and proteins were visualized by enhanced chemiluminescence western blot reagents (Millipore, USA).

### Cell transfection

Negative control (NC), miR-448 inhibitor, miR-448 mimic, si-control, and si-SIRT1 were all purchased from GenePharma Co. Ltd. (China) for cell transfection. The nerve cell lines AGE1.HN and PC12 were seeded in a 6-well plate and transfected with different plasmids by Lipofectamine2000 (Invitrogen) according to the specification. The efficiency of cell transfection was examined by qRT-PCR and western blot analysis.

### Cell apoptosis assay

The Annexin V-FITC/propidium iodide (PI) cell apoptosis detection kit (Sigma, USA) was used to detect nerve cell apoptosis by a flow cytometer FACSCalibur (BD Biosciences, USA). The nerve cell lines AGE1.HN and PC12 (1×10^6^ cells/well) were washed with PBS and stained with Annexin V-FITC and PI for 30 min at 37°C. The stained nerve cells were analyzed by flow cytometry with FACSCalibur (BD Biosciences).

### Dual-luciferase reporter assay

To investigate the regulative relationship between miR-448 and SIRT1, the SIRT1 3′-UTR DNA segments containing the predicted miR-448 binding site were inserted into pmirGLO vector (Promega, USA). The recombinant vectors and NC, miR-448 inhibitor, or miR-448 mimic were co-transfected into HEK-293 cells with Lipofectamine2000 (Invitrogen). Luciferase activity was detected by the dual luciferase reporter assay system (Promega) following the manufacturer's manual.

### Plasmid construction

The pcDNA3.1 was used to establish the pcDNA-SIRT1 expression plasmid, which was transfected into neurons by Lipofectamine2000. Briefly, the target gene was amplified by PCR and the product was purified by gel extraction. Then the purified SIRT1 product and pcDNA3.1 were integrated into pcDNA-SIRT1 recombinant plasmid after double enzymes restriction and linkage with T4 DNA ligase (Takara, Japan). The recombinant pcDNA-SIRT1 was inserted into *E. coli* for selection and amplification of positive clones, which were transfected into neurons after identification with restriction analysis and sequence analysis by Sangon Biotech. Finally, the pcDNA-SIRT1 was transfected into cells by using Lipofectamine2000.

### Spinal cord neurological evaluation

Twenty adult male Sprague Dawley rats were randomly divided into two groups and intrathecally infused with 100 μL of miR-448 inhibitor (n=10) or negative control (NC, n=10) with Lipofectamine2000 (Invitrogen) continuously for 3 days before the ischemia/reperfusion surgery (16). Forty-eight hours after the ischemia/reperfusion surgery, the hind limb motor function was assessed by the BBB scale and the neurological function was evaluated using the motor deficit index (MDI) score according to the ambulation and placing/stepping responses (17), which was performed by a study author who was blind to the groups.

### Statistical analysis

Data are reported as means±SD, and all statistical analyses in this study were carried out by SPSS 18.0 software package (SPSS Inc., USA). Differences between groups were determined by Student's *t*-test and a P value of <0.05 was considered statistically significant.

## Results

### Altered expression of miR-448 and SIRT1 in SCII tissues

**Figure 1.**
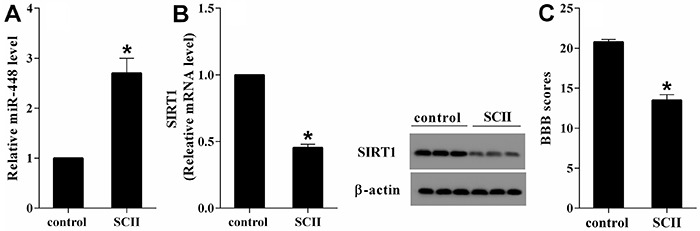
Altered expression of miR-448 and SIRT1 in ischemia/reperfusion injury (SCII) tissues. *A*, Expression of miR-448 in control and SCII tissues was quantified by qRT-PCR (n=12). *B*, Expressions of SIRT1 mRNA and protein were analyzed by qRT-PCR and western blot (n=12), respectively. *C*, Hind limb motor function was assessed by Basso, Beattie, and Bresnahan (BBB) scoring. Data are reported as means±SD. *P<0.05 *vs* control (*t*-test).

Compared with the control group (n=12), the expression of miR-448 in SCII tissues (n=12, SCII) was significantly increased (Figure 1A), while the expressions of SIRT1 mRNA and protein were dramatically decreased in SCII tissues (Figure 1B). The BBB score in SCII experimental group was markedly lower than that of control group, suggesting that the hind-limb motor function of the SCII rat was significantly affected (Figure 1C).

### Hypoxia treatment altered the expression of miR-448 and SIRT1 in nerve cells

**Figure 2.**
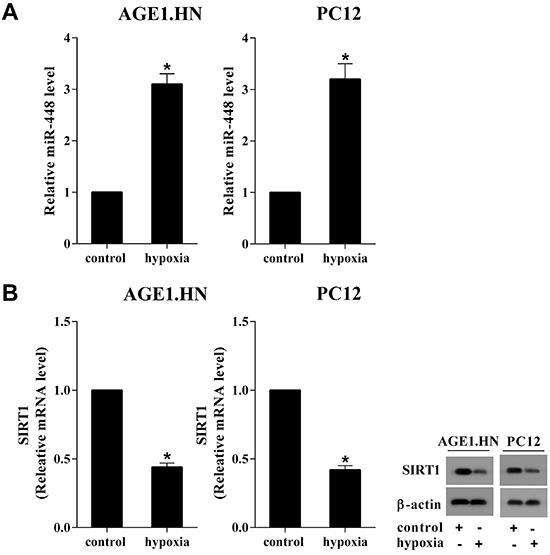
Hypoxia treatment altered the expression of miR-448 and SIRT1 in nerve cells. *A*, Expression of miR-448 in AGE1.HN and PC12 cells with or without hypoxia treatment was determined by qRT-PCR. *B*, Expressions of SIRT1 mRNA and protein in AGE1.HN and PC12 cells with or without hypoxia treatment were analyzed by qRT-PCR and western blot, respectively. Data are reported as means±SD. *P<0.05 *vs* control (*t*-test).

Compared with the control group, the expression of miR-448 was up-regulated in AGE1.HN and PC12 cells after hypoxia treatment (Figure 2A), while expression of SIRT1 mRNA and protein exhibited a significant drop after hypoxia treatment (Figure 2B).

### Hypoxia treatment down-regulated SIRT1 through up-regulating miR-448

**Figure 3.**
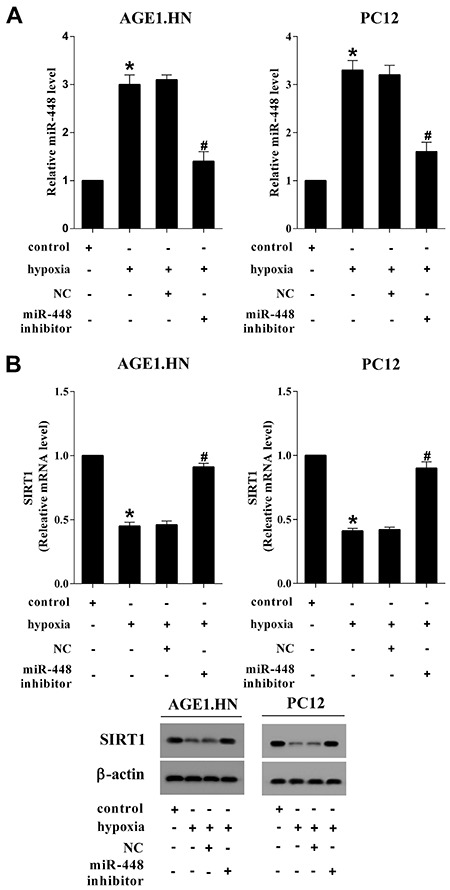
Hypoxia treatment regulated the expression of SIRT1 through miR-448. *A*, Expression of miR-448 in AGE1.HN and PC12 cells in control or treated with hypoxia, or transfected with NC or miR-448 inhibitor was determined by qRT-PCR. *B*, Expressions of SIRT1 mRNA and protein in AGE1.HN and PC12 cells in control or treated with hypoxia or transfected with NC or miR-448 inhibitor were analyzed by qRT-PCR and western blot, respectively. Data are reported as means±SD. *P<0.05 *vs* control. ^#^P<0.05 *vs* NC (*t*-test). NC: negative control.

Results showed that hypoxia treatment up-regulated miR-448 expression, which was reversed by miR-448 inhibitor (Figure 3A), while the hypoxia treatment down-regulated the SIRT1 expression, which was reversed by miR-448 inhibitor (Figure 3B). These findings indicated that hypoxia treatment down-regulated SIRT1 by up-regulating miR-448.

### Overexpression of miR-448 promoted nerve cell apoptosis

**Figure 4.**
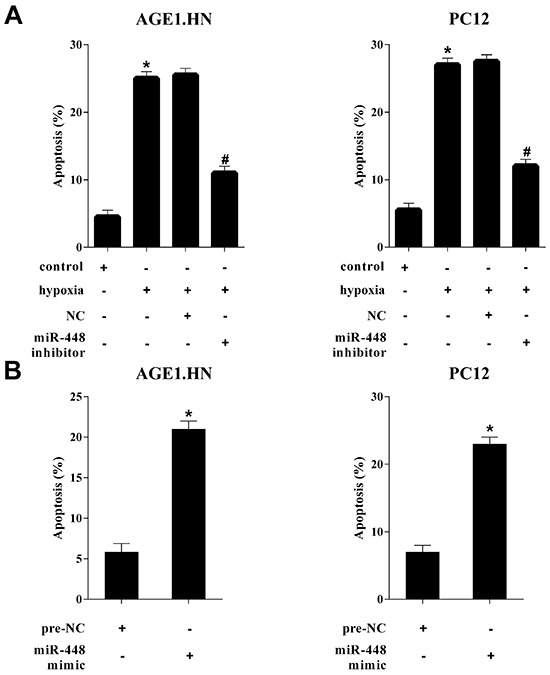
MiR-448 participated in the regulation of nerve cell apoptosis. *A*, Cell apoptosis was analyzed by flow cytometry in AGE1.HN and PC12 cells treated with hypoxia, or transfected with NC or miR-448 inhibitor. *P<0.05 *vs* control. ^#^P<0.05 *vs* NC (*t*-test). *B*, Cell apoptosis was analyzed by flow cytometry in AGE1.HN and PC12 cells transfected with pre-NC or miR-448 mimic. Data are reported as means±SD. *P<0.05 *vs* pre-NC (*t*-test). NC: negative control.

Cell apoptosis level was detected in the condition of hypoxia treatment. Results showed that cell apoptosis was clearly promoted by hypoxia treatment, which was reversed by miR-448 inhibitor (Figure 4A). Under normal conditions, miR-448 was overexpressed in AGE1.HN and PC12 cells by transfecting miR-448 mimic, and apoptosis was significantly promoted (Figure 4B).

### Expression of SIRT1 was regulated by miR-448 in AGE1.HN cells

**Figure 5.**
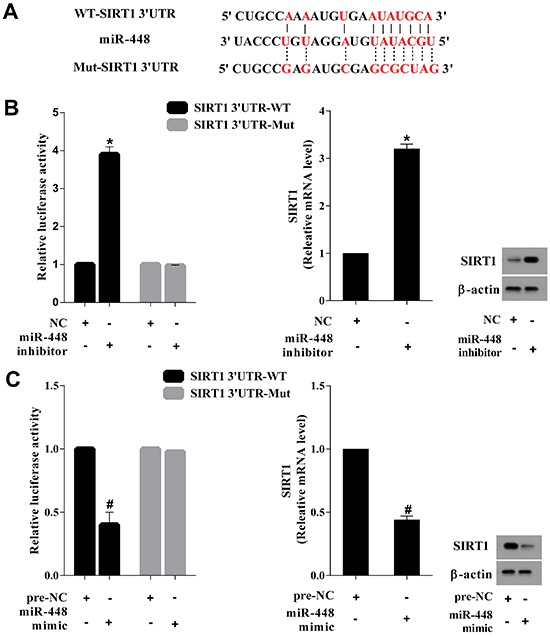
Expression of SIRT1 was regulated by miR-448 in AGE1.HN cells. *A*, Predicted binding region of miR-448 and the 3′-UTR of WT-SIRT1, and sequence of the mut-SIRT1 3′-UTR. *B*, Relative luciferase activity of WT-SIRT1 and mut-SIRT1 in AGE1.HN cells transfected with NC or miR-448 inhibitor. Expressions of SIRT1 mRNA and protein in AGE1.HN cells transfected with NC or miR-448 inhibitor were analyzed by qRT-PCR and western blot, respectively. *P<0.05 *vs* NC (*t*-test). *C*, Relative luciferase activity of WT-SIRT1 and mut-SIRT1 in AGE1.HN cells transfected with pre-NC or miR-448 mimic. Expressions of SIRT1 mRNA and protein in AGE1.HN cells transfected with pre-NC or miR-448 mimic were analyzed by qRT-PCR and western blot, respectively. Data are reported as means±SD. ^#^P<0.05 *vs* pre-NC (*t*-test). NC: negative control.

The binding region of miR-448 and the 3′-UTR of WT-SIRT1 was predicted by bioinformatics analysis (TargetScan and microrna.org; Figure 5A). Compared with NC, inhibition of miR-448 improved the activity of WT-SIRT1 3′-UTR, and it up-regulated SIRT1 mRNA and protein; no difference in SIRT1 3′UTR-mut activity between the two groups was observed (Figure 5B). Compared with Pre-NC, overexpression of miR-448 with miR-448 mimic suppressed the activity of WT-SIRT1 3′-UTR, and it down-regulated SIRT1 mRNA and protein; no significant difference in SIRT1 3′UTR-mut activity between the two groups was noted (Figure 5C).

### Hypoxia treatment down-regulated SIRT1 to promote nerve cell apoptosis by up-regulating miR-448

**Figure 6.**
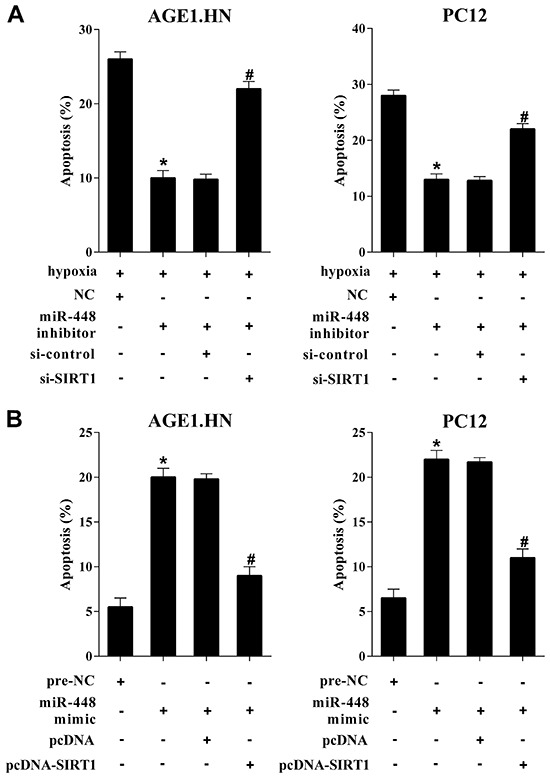
Hypoxia treatments regulated the expression of SIRT1 through miR-448 for participating in nerve cell apoptosis. *A*, Cell apoptosis analyzed by flow cytometry in AGE1.HN and PC12 cells treated with hypoxia, and transfected or co-transfected with NC or miR-448 inhibitor or si-control or si-SIRT1. *P<0.05 *vs* NC. ^#^P<0.05 *vs* si-control (*t*-test). *B*, Cell apoptosis analyzed by flow cytometry in AGE1.HN and PC12 cells transfected with pre-NC or miR-448 mimic. Data are reported as means±SD. *P<0.05 *vs* pre-NC. ^#^P<0.05 *vs* pcDNA (*t*-test). NC: negative control.

In AGE1.HN and PC12 cells, hypoxia treatment significantly facilitated apoptosis, which was dramatically reversed by miR-448 inhibitor, while the effect was further reversed by si-SIRT1 (Figure 6A). Under normal conditions, with miR-448 overexpressed in AGE1.HN and PC12 cells by transfecting miR-448 mimic, apoptosis was clearly promoted, which was reversed by pc-DNA-SIRT1 (Figure 6B). It showed that up-regulation of miR-448 induced by hypoxia promoted apoptosis of nerve cells by down-regulating SIRT1.

### Impact of miR-448 on neurological function and motor function of rats with SCII

**Figure 7.**
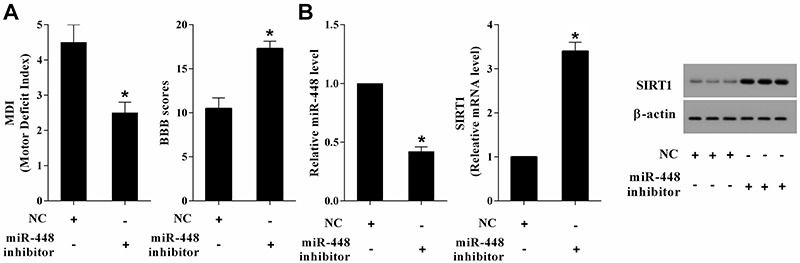
Impact of miR-448 expression level on neurological function of rats with ischemia/reperfusion injury (SCII). *A*, Motor deficit index (MDI) score and the Basso, Beattie, and Bresnahan (BBB) score. *B*, Expressions of miR-448, SIRT1 mRNA and protein in spinal tissues transfected with NC (n=10) or miR-448 inhibitor (n=10) analyzed by qRT-PCR and western blot, respectively. Data are reported as means±SD. *P<0.05 *vs* NC (*t*-test). NC: negative control.

Rats were considered without paraplegia if the MDI score was <3 and with paraplegia if MDI ≥3; hind limb motor function was assessed by BBB scoring. The results showed that miR-448 inhibitor clearly decreased the MDI score and increased the BBB score, suggesting a neuroprotective effect on adult rats (Figure 7A). Compared with NC, the expression of miR-448 in spinal tissue was decreased while the expression of SIRT1 was increased by miR-448 inhibitor (Figure 7B).

## Discussion

In general, SCI refers to primary mechanical damage in spinal cord and secondary damage caused by subsequent biological processes such as inflammation, oxidation, apoptosis, and altered gene expression (18). Distinctively, SCII is usually caused by a thoracoabdominal aortic surgery, but it evokes similar complications with SCI that afflict thousands of individuals and have been receiving numerous attentions. This study showed high expression level of miR-448 and low expression level of SIRT1 in SCII tissues, implying a connection between expression levels of miR-448 and SIRT1 with SCII development. We showed that high expression of miR-448 down-regulated SIRT1 and further promoted apoptosis of nerve cells. Down-regulated miR-448 inhibits apoptosis of nerve cells and improves neurological function by up-regulating SIRT1, which contributes to relieving SCII of rats.

The spinal cord is a rich source of miRNAs, and those whose temporal expression was altered following SCI were classified into three types: miRNAs with up-regulated expression, miRNAs with down-regulated expression, and those with an initial up-regulation and then down-regulation after SCI (19). Several studies have reported that miRNAs altered the response to cerebral ischemia reperfusion injury by regulating the expression of various key elements in cell growth and apoptosis (20). MiR-497 has been proved to inhibit inflammation and apoptosis of SCII through its targets, IRAK1 of TLR4 and Cyclic AMP response element binding protein (CREB) signaling pathway (21). By using the SCII model in our study, we revealed that expression of miR-448 was dramatically increased in the SCII tissues and it promoted apoptosis, implying its key role in SCII progression. Changes in miRNA expression participate in numerous biological processes in SCII physiopathology such as inflammation, demyelination, apoptosis, and regeneration. A previous study also has demonstrated that morphine decreased expression of miR-448 in hippocampus of stressed neonatal mice, implying the significant influence of miR-448 expression on central nervous system change (22). Defining the underlying mechanism of the miRNA up-regulation induced by SCII was complicated due to the heterogeneity of spinal cord cell and multiple changes that occurred after SCII. But miRNA expression appeared to be influenced by its expression specificity in spinal cord cells and the invasion of immune cells at the injury site (23,24). Ischemia is followed by hypoxia, which causes tissue and vascular damage. Up-regulation of miR-448 caused by hypoxia was shown in this study, which may be achieved by altering the biogenesis, processing, maturation, or degradation of miR-448 (25).

**Figure 8.**
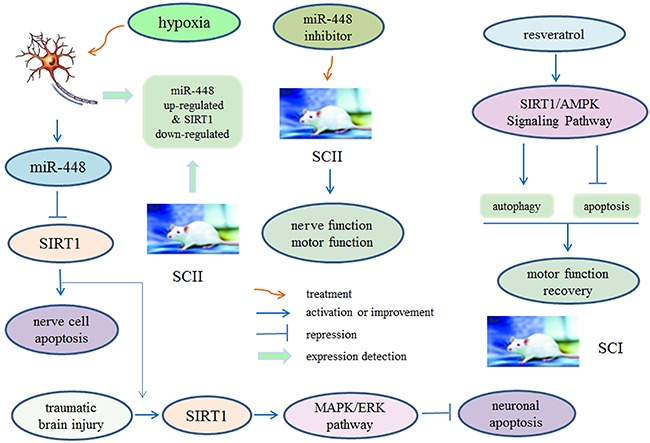
Flowchart illustrating the different findings reported on the neuroprotective effect of SRIT1 by suppressing neuronal apoptosis in ischemia/reperfusion injury (SCII). SCI: spinal cord injury.

The neuroprotective effect of SIRT1 has been discussed previously in EAE (6), and it has also been reported that SIRT1 expression and activity are up-regulated in the brain tissue of epileptic patients and rat models (26). Up-regulation of SIRT1/AMPK signaling pathway after SCI was involved in the anti-apoptosis effect of resveratrol; namely, resveratrol suppressed apoptosis by up-regulating the SIRT1/AMPK pathway and thereby promoting motor function recovery and motor neuron survival (27). The inhibitory effect of SIRT1 on neuronal apoptosis in central nervous system changes has been highlighted, which may be achieved by activating the MAPK/ERK pathway (28) (Figure 8). Findings of the current study were in accordance with these previous studies, and decreased expression of SIRT1 in SCII tissues implied its pivotal role in pathogenesis of SCII. Up-regulation of SIRT1 by decreased miR-448 inhibited apoptosis of nerve cells and improved neurological function of rats with SCII, manifesting the neuroprotective role of SIRT1 following SCII. With motor and nerve function evaluated, we reconfirmed the protective effect of SIRT1 in facilitating recovery after SCII. Although the underlying mechanisms about modulating apoptosis and improving neurological function of SIRT1 in SCII remain to be elucidated, research on potent SIRT1 agonist SRT1720 is already under way (29), which may be a feasible therapeutic agent for clinical application in SCII.

This study is the first to investigate the relationship of miR-448 and SIRT1 following SCII, and it confirmed that down-regulated miR-448 inhibited apoptosis of nerve cells and improved neurological function by up-regulating SIRT1, which contributed to alleviating SCII. This study provides a novel and promising molecular mechanism for SCII therapy, and the expressional changes in miR-448 and the corresponding clinical manifestations may offer references for future clinical studies on miRNAs. Further study on miRNAs in SCII is urgently needed to develop effective and safe therapeutic strategies for patients with SCII, and to improve the prognosis of SCII.
